# Disagreement, Certainties, Relativism

**DOI:** 10.1007/s11245-018-9567-z

**Published:** 2018-06-22

**Authors:** Martin Kusch

**Affiliations:** Department of Philosophy, University of Vienna, Universitätsstrasse 7, 1090 Vienna, Austria

**Keywords:** Disagreement, Peer disagreement, Certainties, Wittgenstein, Relativism

## Abstract

This paper seeks to widen the dialogue between the “epistemology of peer disagreement” and the epistemology informed by Wittgenstein’s last notebooks, later edited as On Certainty. The paper defends the following theses: (i) not all certainties are groundless; many of them are beliefs; and they do not have a common essence. (ii) An epistemic peer need not share all of my certainties. (iii) Which response (steadfast, conciliationist etc.) to a disagreement over a certainty is called for, depends on the type of certainty in question. Sometimes a form of relativism is the right response. (iv) Reasonable, mutually recognized peer disagreement over a certainty is possible.—The paper thus addresses both interpretative and systematic issues. It uses Wittgenstein as a resource for thinking about peer disagreement over certainties.

## Introduction

In this paper I want to widen the dialogue between the *epistemology of peer disagreement* and so-called *hinge epistemology*, that is, the epistemology informed by Wittgenstein’s last notebooks, later edited as *On Certainty* (Wittgenstein 1969). In doing so I continue a line of research started more than thirty years ago in Robert J. Fogelin’s little classic “The Logic of Deep Disagreement” (1985).^1^ I shall address the following questions: What are (Wittgensteinian) certainties? Can we rationally count someone a peer who denies one of our certainties? Can there be reasonable, mutually recognized, peer disagreements over a certainty? And finally, faced with a peer disagreement over a certainty (= *c-disagreement*), should I be “steadfast” (Kelly 2005), “conciliationist” (Christensen 2007), “justificationist” (Lackey 2008), or “relativist” (Hazlett 2014)?

In answer to these questions, I shall try to make plausible, in a preliminary and tentative fashion, the following theses:

(i) Not all certainties are groundless; many of them are beliefs; and they do not have a common essence.
(ii) The analysis of different forms of peer disagreement calls for different renderings of the category of peer: *ordinary peer, distant peer, scientific peer*, and *stance-dependent peer*. As we shall see, this distinction allows us to maintain that an epistemic peer need not share all of my certainties.
(iii) Which responses (steadfast, conciliationist etc.) to c-disagreements are called for, depends on the types of certainty in question. Some c-disagreements call for (different) forms of relativism pace Feldman (2011). I shall speak of *cultural relativism, relativism of reasonable belief, epistemic relativism*, and *stance relativism*.
(iv) Reasonable, mutually recognized peer c-disagreement is possible.

In other words, my analyses suggest more than one form of response to a c-disagreement; more than one rendering of the category of peer; and more than one understanding of relativism. No doubt it would have been more elegant to arrive at a less complex account of c-disagreements, but this is what Wittgenstein and the data suggest. Note that the paper is thus addressing both interpretative and systematic issues. Wittgenstein is used as a resource for thinking about peer disagreement over certainties more generally.

Lest some readers will be disappointed by the sketchy and programmatic nature of what follows, it is perhaps worth saying that this study is more about offering a preliminary exploration of an area of investigation than about fine conceptual distinctions or detailed defences against all conceivable objections. As Kant once inimitably put it in defence of the intricacy and complexity of his epistemological writings: “Hammer and chisel are perfectly fine for working raw lumber, but for copperplate one must use an etching needle” (Kant 2004, p. 9). As far as c-disagreements are concerned, we are still in the era of raw lumber; the time of copperplate and needlework is still to come.

## Certainties

This paper has two main parts. I begin by outlining the understanding of certainties or hinges that I have developed and defended in a more than a dozen papers over the last decade.^2^ Subsequently I suggest some ways in which one might bring my rendering of certainties to bear on the issue of peer disagreement. In so doing, I am assuming—without further argument—that my interpretation of certainties is correct. This is of course a highly contentious assumption. But there is only so much one can do in one paper. I hope that the reader will either consider the evidence offered in my earlier studies, or else be willing to consider the arguments of this paper in a conditional way: what would follow for peer disagreement, if the present paper were right about the interpretation of certainties?

On the reading offered here, the position *On Certainty* is anxious to reject divides our system of beliefs into two components: semantic and mathematical beliefs are the foundation and beyond doubt; empirical beliefs are not ultimately foundational and always in principle doubtful:

I am no more certain of the meaning of my words than I am of certain [empirical] judgments. … (1969, §126)

… If one doesn’t marvel at the fact that the propositions of arithmetic (…) are ‘absolutely certain’, then why should one be astonished that the proposition ‘This is my hand’ is so equally? (1969, §448)

These passages suggest the following view. Some empirical beliefs (or empirical judgments) can, in at least some contexts, be as foundational as are beliefs about the meanings of words or mathematical results. And not all beliefs about the meanings of words or mathematical results are as certain as are at least a subset of empirical beliefs. Moreover, *On Certainty* is best read as allowing, first, that most—but not all—certainties are propositional, and supported or supportable by epistemic evidence; and, second, that all certainties are sometimes used as standards or rules for the formation of other beliefs. All of them are, as Wittgenstein writes in §674: “Cases in which I rightly say I cannot be making a mistake …” Wittgenstein continues: “I can … enumerate various typical cases, but not give any common characteristic.” This comment should be taken seriously. It indicates that for Wittgenstein there is no succinct definition of certainties. Certainties form something of a family, with various similarities between different members, but no overall common feature except for the “cases in which I rightly say I cannot be making a mistake”. In the following I shall try to put together a list of typical cases. I propose a preliminary classification of 11 types on the basis of what the respective certainty is about. These 11 types fall into five distinct epistemic *categories*.

Category I cases are beliefs for which we typically have overwhelming evidence that is difficult to “export” or “share” all at once. There are seven types of this category (Wittgenstein 1969):

- Beliefs about one’s own body (e.g., “… here is a hand.” §1)
- Perceptual beliefs about close familiar medium-size objects (e.g., “I believe there is an armchair over there.” §193)
- Introspective beliefs (e.g., “I am in pain.” §178)
- Memory beliefs of salient features of one’s autobiography (e.g., “I have lived in this room for weeks past.” §416)
- Simple inductive beliefs, e.g., about familiar simple objects (“After putting a book in a drawer, I assume it is there ...” §134)
- Testimonial beliefs based on textbook testimony (“… textbooks of experimental physics … I trust them.” §600)
- Semantic beliefs (e.g., “My name is L.W.” §425)

Category II cases are beliefs beyond direct evidential support; they are beliefs that constitute domains of knowledge. An example is: “... the earth exists” (§209).

Category III consists of certainties based on overwhelming evidence that supports at least a (temporary, ‘for the time being’) immunization against possible refutation. These certainties are fundamental empirical-scientific beliefs or, as I shall say, “cornerstones of scientific paradigms”. For instance, “Water boils at 100 degrees Celsius.” (§291). Note that Wittgenstein is happy to speak of knowledge here: “We know that the Earth is round” (§291).

Category IV are certainties based on overwhelming evidence that support a once-and-for-all immunization (against possible empirical refutation). These are mathematical propositions that have “officially, been given the stamp of incontestability …” (§655).

Finally, Category V are fundamental religious beliefs. I shall later interpret them as certainties tied to a “stance” and supported primarily by stance-internal evidence. An example is: “Jesus only had a human mother” (§239).

In the rest of my paper, I shall go through examples of each of these five categories and investigate, in a preliminary fashion, what kinds of disagreements over the respective certainties are possible.^3^

## Peer Disagreements Over Different Types of Certainties

### Category I: Beliefs About One’s Own Body

I begin with beliefs about my own body that I cannot easily be wrong about. Take my belief, B1, that I have two hands. Do I have evidence and justification for B1? The answer to this question depends on how one understands evidence and justification. Assume that the understanding defended by Goldman since the 1980s (e.g., Goldman 1980) is at least roughly on the right track. According to this “historical” conception, a belief can remain justified even though the evidence for it has largely been forgotten. Given this rendering of evidence and justification, it seems obvious that B1 *is* justified. Note also that even if I were able to hold all of my evidence for B1 in my mind simultaneously, I would still be unable to fully share it with others. This is because part of the evidence consists of *my* experiences: I can describe these experiences to others, but I cannot literally let these others *have* my experiences. Plausibly we can render this observation by saying that most of us give B1 a much higher credence than is epistemically justified in terms of evidence here and now. Perhaps one can even maintain that most of us have long since stopped updating B1 on our sensory evidence. It is for these reasons—that most of the evidence is forgotten, that one cannot easily share it with others, and that one has stopped collecting it—that the evidence for beliefs like B1 cannot always play a straightforward role in “dialectical justification”, that is, the kind of justification we offer in response to challenges. And yet, on the historical conception of evidence all this does not make B1 groundless.

It does not undermine this interpretation that in the context of his criticism of G. E. Moore’s “Proof of an External World” (Moore 1939), Wittgenstein denies that B1 is knowledge-apt (1969, §§1–10). On the one hand, Wittgenstein seems to have wavered on this question; the mentioned early passages seem to deny that B1 is knowledge-apt, while later passages explicitly allow for it (1969, §§397, 480, 552). And, on the other hand, Wittgenstein nowhere argues that B1 is not knowledge-apt since it is not rationally held. The real problem with Moore’s self-attribution of knowledge concerning B1 is that Moore takes this self-attribution to refute the skeptic and idealist. As Wittgenstein sees it, the skeptic’s or idealist’s doubts are not of a kind that can be refuted by self-attributions of knowledge. Moore conflates practical, pedestrian doubt with radical doubt. Put differently, the skeptic and idealist do not deny our sensory or experiential data, but they refuse to interpret this data as we do, that is, as evidence for a mind-external world. According to Wittgenstein, self-attributions of knowledge cannot be used to establish one rather than another evidential interpretation of the sum total of our data (1969, §19).

In speaking of the “evidential interpretation of … our data” I am using an idea familiar from the philosophy of science and recently introduced into the peer-disagreement literature by Decker (2012). That is to say, we should distinguish between *data* and *evidence*: evidence is data interpreted in such a way that it can give support to a given belief. The idea, it seems to me, has a natural application to Wittgenstein’s analysis of what goes wrong between Moore and the skeptic. Moore and the skeptic have roughly the same data (that is, the same sensory appearances), but whereas Moore turns this data into evidence for his realism about his hand, the skeptic refuses to do so based on his skeptical (idealist or phenomenalist) interpretational scheme.

I have not yet directly addressed the question whether there can be a peer disagreement over B1 (say, uttered by me). Let us take the category of peer here in an “ordinary” sense. By “ordinary peer” I mean someone who, in my estimation, has roughly the same “general epistemic virtues such as intelligence, thoughtfulness, and freedom from bias” as I do (Kelly 2005, p. 175). If ‘peer’ is taken in this way, then over a very wide range of contexts such a peer disagreement over B1 seems impossible. The reason for this is that our evidence for B1 is much larger than our evidence for either the belief that the disagreement is real (and not just verbal), or that the person disagreeing with us is our ordinary peer when it comes to questions concerning our handedness. In other words, when an interlocutor denies B1 (as uttered by me), the rational response for me is to either assume that he does not understand my words, or suspect that he is not my intellectual peer.

Does this rule out a skeptical challenge concerning B1? Is it possible to rationally disagree with the skeptic? My use of Decker (2012) might seem to support a positive reply: after all, Decker allows for rational disagreement after full disclosure of data, provided that the cause of the disagreement lies with the employment of different evidential interpretations. And this is precisely the way I couched the relationship between Moore and the skeptic above. This issue is best addressed in the context of my next section.

### Category II: Beliefs that Constitute Domains-of-Knowledge

The paradigmatic example of this category is (B2): “... the earth exists” (1969, §209). At one point Wittgenstein writes that B2 is “part of the whole picture which forms the starting-point of belief for me” (§209). It is unclear whether this is meant to suggest that strictly speaking B2 is no a belief or not even propositional. This suggestion would fit with Daniele Moyal-Sharrock’s proposal that B2 is best taken as an element in a Searlean “background” (2007, p. 61). If this is the correct view, then there can be no question of the skeptic disagreeing with us: there would be no difference in beliefs or judgments.

There are, however, also places that suggest a propositional rendering of “the earth exists”. For instance, Wittgenstein writes that beliefs like B2 are “foundation walls … carried by the whole house” (1969, §248). And it seems natural to understand this idea in light of §410: “Our knowledge forms an enormous system. And only within this system has a particular bit the value we give it.” In other words, B2 is justified by belonging to a coherent system of beliefs. Wittgenstein also remarks that B2 is, and has always been, universally shared: “for unthinkable ages, it has belonged to the scaffolding of our thoughts” (1969, §211). Thus someone who denies B2, or our knowledge of it, has an overwhelming “consensus gentium” against him or her.

What follows from this for the peer-status of the skeptic? Perhaps the most natural thing to say is that Wittgenstein wants to deny the skeptic such a status. The skeptic simply lacks an appropriate understanding of our epistemic reasoning. And this is a lack of epistemic virtue. In other words, and to cut a long story short, Wittgenstein thinks epistemic skepticism is based on a lack of appreciation of, amongst others, the ideas that our beliefs in general and our certainties in particular are parts of a shared system of beliefs (1969, §§410, 211); that doubts have to be earned (1969, §337); that doubts presuppose something which is not doubtful (1969, §337); or that external-world skepticism collapses into self-refuting semantic skepticism (1969, §§317, 369, 456, 507). We thus cannot but demote the skeptic, or we should not take him to be an ordinary epistemic peer in the first place. In order to qualify as an ordinary epistemic peer, Wittgenstein thinks, an interlocutor must not only be intelligent and familiar with the data; he or she must also have a correct understanding of our epistemic practices. And the skeptic lacks precisely such understanding.

### Category III: Mathematical Propositions

At issue here, it will be recalled, are beliefs about elementary arithmetic (1969, §43) that have “been given the stamp of incontestability ...” (1969, §655). It is important to remember that for Wittgenstein a mathematical proof is a one-time experiment the outcome of which we treat as a norm for other experiments of the same kind: “We might have adopted 2 + 2 = 4 because two balls and two balls balances four. But now we adopt it, it is aloof from experiment—it is petrified” (Wittgenstein 1976, p. 98). That is to say, at first we have very strong and compelling empirical evidence for two times two balls equaling four balls in weight. But for this experiment to function as a proof we have to go further and immunize it against all possible empirical refutation. And we do so for the “greatest variety of reasons” (2000, p. 122, 58v–59r).

Can there be peer disagreement concerning simple calculations? Not for someone who we have reason to assume intends to calculate according to our mathematics. Nor for someone who declares all our calculations “uncertain”: “perhaps we would say he was crazy” (1969, §217). And yet, Wittgenstein does allow for alternative and complete forms of arithmetic: “Primitive arithmetic is not incomplete, even one in which there are only the first five numerals …” (2001, pp. 101–2). Wittgenstein never discusses an example of such an alternative arithmetic in any detail. But he seems to think that the “Odd Woodsellers” are akin to people with a different arithmetic. At least this is how this example is introduced in Wittgenstein’s writings on the philosophy of mathematics. The Odd Woodsellers sell wood by area covered, disregarding the height of the piles. Are they our mathematical peers? Do we even have a case of disagreement here?

Wittgenstein makes four observations regarding this fictitious tribe. The first observation is that if we are unable to convince the Odd Woodsellers of the equality in value of two piles (same number of logs, different area covered) by re-arranging the piles, then we “should presumably say … they simply do not mean the same by ‘a lot of wood’ and ‘a little wood’ as we do …” (1976, p. 202). Note the cautious way in which Wittgenstein puts the remark: “we should presumably say”. Perhaps this is informed by his general reluctance to legislate on whether two speakers mean the same or different things by the same words. I am thinking here of Wittgenstein’s discussion of a measuring practice based on soft and expanding rulers: “It can be said: What is here called ‘measuring’ and ‘length’ and ‘equal length’, is something different from what we call those things. The use of these words is different from ours; but it is akin to it; and we too use these words in a variety of ways” (1978, p. I 5).

Wittgenstein’s second observation is that we could easily *exploit* the Odd Woodsellers. We could say: “I’ll give you my Pile B for your Pile A” (where B covers the same area but has fewer logs than A). But that *we* could exploit the Odd Woodsellers does not mean that they would have to exploit each other. If they do not, then their practice might well be functional in their society (1976, p. 202).

Third, Wittgenstein wonders whether the Odd Woodsellers suffer from the “logical madness” that plays a central role in Frege’s criticism of psychologism. Again the response defends the practice: “We might call this a kind of logical madness. But there is nothing wrong with giving wood away. So what is wrong with this? We might say, ‘This is how they do it.’” (1976, p. 202).

Finally, fourth, Wittgenstein reflects on the question whether the practice of the Odd Woodsellers would not be altogether “pointless”. And his response is this: “Pointless? Well, much is pointless in our culture, too. Think of coronations!” (1978, p. I 150). (Wittgenstein was obviously not impressed with the coronation of King Georg IV on 12 May 1937.)

The upshot of these remarks is that while the Odd Woodsellers’ way of measuring the value of wood is very different from ours, there is no knock-down argument against their practice. Note what Wittgenstein is doing here: he rescues the Odd Woodsellers’ rationality by removing the appearance of disagreement. For instance, in suggesting that they mean something different by “little wood” than we do, he treats the difference between them and us in a contextualist fashion. Wittgenstein is using a “principle of charity”: he is keen to preserve the (mathematical) rationality of the tribesmen. That is, in a situation where the aim is to give away wood for free, it might be perfectly rational to treat the two piles (of different number of logs, but same area covered) as having the same value. Naturally, *our* institution of mathematics is not part and parcel of an institution of gift-giving. The Odd Woodsellers are thus right only in their context; we are right in our context. Maybe we could describe the situation using the term “distant peers” (a term introduced by Vorobej (2011) for a different idea): Wittgenstein suggests that the Odd Woodsellers act as we would do, if we were placed in their social setting. There is also a whiff of *cultural relativism* in Wittgenstein’s discussion: cultures and their institutions cannot be ranked, in a neutral and universal fashion, as more or less rational. There is no higher court of appeal that would decide the disagreement between us and the Odd Woodsellers. They may well be right in their context, and we are right in ours. Neither we nor they are *absolutely* right or *absolutely* wrong. Note that such a relativist response has elements of both the steadfast and the conciliationist positions: steadfast insofar as one is entitled to retain one’s position, despite the disagreement; conciliationist insofar as one does not count oneself uniquely or absolutely right (Kusch 2016a).^4^

### Category IV: Cornerstones of Scientific Paradigms

Wittgenstein gives a number of examples of certainties belonging to the natural sciences. Especially interesting is the following certainty that he ascribes to Lavoisier: “a substance A always reacts to a substance B in the same way, given the same circumstances” (1969, §168). Maybe this is meant to be the principle of the uniformity of nature. But the fact that Wittgenstein attributes this principle specifically to Lavoisier suggests that he might have had something different in mind, namely Lavoisier’s “compositionism”, a principle that contrasts with Priestley’s and other phlogistonists’ “principlism” (Siegfried 1989; Chang 2012; Kusch 2015b). According to Lavoisier’s compositionism, chemical substances divide into (equally fundamental and ponderable) “elements” and ponderable “compounds”. For the principlist Priestley, chemical reality consists of imponderable fundamental principles (like phlogiston) and passive substances. As Thomas Kuhn famously argued, Lavoisier and Priestley worked within different “paradigms” (Kuhn 1962). And different paradigms are based on different “cornerstone beliefs”, such as compositionism and principlism. I am here following Georg Henrik von Wright (1982) who once suggested that it might be fruitful to relate Kuhn’s paradigms to Wittgenstein’s certainties.

Was the disagreement between Priestley and Lavoisier a peer disagreement? The case seems worthy of investigation not least because scientific disagreements are never discussed in the peer-disagreement literature. Consider first the role of principles such as compositionism or principlism. Clearly, they are not directly based on evidence; they are—in Decker’s sense introduced above—interpretational schemes for turning empirical data into evidence for paradigm-specific claims. Second, did Lavoisier and Priestley think of each other as epistemic peers? That depends on how we define the term. They certainly did not think of each other as being equally likely to be right about chemical matters. And yet, they did regard each other as serious scientists; they learnt from each other; used each other’s data; and felt it was possible and desirable to convince the other. I propose using the label ‘scientific peers’ for this status. Third, did Lavoisier and Priestley fully disclose all of their evidence, or, more precisely, did they disclose all of their data? There is some scholarly dispute on this issue (Kusch 2015b), but the most recent and much-discussed study by Chang (2012) claims that they did. And this brings us, fourth, to the central question: What should Lavoisier and Priestley have done when faced with their scientific peer disagreement after full disclosure? Chang’s answer is based on his “scientific pluralism”: Lavoisier and Priestley should have counted each other as epistemically blameless; as wrong; and as entitled to stick to their guns.

Chang’s scientific pluralism is the equivalent, in the philosophy of science, of a position that, working in epistemology, Hazlett (2014) has dubbed “naïve liberalism”; that is, the view that there can be “reasonable, mutually recognized peer disagreement”. Cutting Hazlett’s much longer story short, and reformulating his criteria for the case of science, one would have to say the following: Priestley and Lavoisier were entitled to stick to their paradigms since by giving up their respective “cornerstone beliefs” (principlism or compositionism), they would have lost a significant area of apparent truths; since neither side had strong evidence suggesting that their respective methodology and theories were unsuccessful; and since neither side had viciously avoided acquiring evidence relevant for assessing their methodology and theories.

Is this a form of epistemic relativism? Hazlett says “yes”. This is because—after full disclosure—two beliefs, that contradict each other, can both be counted as reasonable. Call this a *relativism of reasonable belief*. At first sight, this relativism seems rather tame. It is tantamount to what the epistemology of disagreement calls “permissiveness”: the idea that with respect to one and the same body of evidence different cognitive responses (belief, disbelief, or suspension of belief) are rationally permissible. Relativism of reasonable belief seems tame, since it is restricted to reasonable belief—not knowledge—and since it is based on the assumption that the conflicting parties are basing their judgments on *one and the same* conception of reasonableness.

Nevertheless, the impression that this relativism is tame in our case is deceptive. To see why consider that, from Lavoisier’s perspective, Priestley is entitled to principlism. Moreover, to be entitled to principlism is to be entitled to much more than just this one principle: it is to be entitled to the whole system of principlist (phlogistonist) chemistry. After all, principlism is the cornerstone of this system. And the scientific rationality that is, as it were, encoded by the phlogistonist system is different from the scientific rationality encoded in Lavoisier’s paradigm. We thus do, after all, have a clash between two forms of epistemic rationality. And this is tantamount to a much more radical form of epistemic relativism, a version that is analyzed and criticized in, say, Boghossian’s work (e.g., 2006). Boghossian’s epistemic relativist is committed to a plurality of incompatible but “equally valid” “epistemic systems” (each with the distinct “fundamental” and “derived” “epistemic principles”).

Admittedly, the bulk of this section went way beyond Wittgenstein’s wording. I have tried to develop his vague reference to Lavoisier’s fundamental commitment into a substantive position that at least resonates with the spirit, if not the letter, of *On Certainty*. To sum up, if I am right about Wittgenstein referring to Lavoisier’s compositionism; if Chang is right about the Chemical Revolution; and if Hazlett is right about naïve liberalism; then disagreements over scientific cornerstone beliefs license a relativistic response.

### Category V: Fundamental Religious Beliefs

The central example in *On Certainty* for this epistemic category is “Jesus only had a human mother” (1969, §239). But again it is advisable to go beyond Wittgenstein’s last notebooks and consider a wider range of texts. As far as Wittgenstein himself is concerned, it seems especially important to incorporate the discussion of his 1938 “Lectures on Religious Belief” (1966). There the central example is (B3): “There will be a Last Judgment.”

Wittgenstein (1966) claims not to have B3 but hesitates to speak of a disagreement between himself and the believer in B3:

Do you contradict the man? I’d say: “No.” ... (1966: 53)

/Do you/... believe the opposite ... ? “... not at all, or not always.” (1966: 53)

Wittgenstein calls B3 “the culmination of a form of life” and an “extraordinary” belief (1966, pp. 58–59). This means that B3 is: a dogma or faith, not a hypothesis (1966, p. 57); linked to strong emotions and pictures (1966, p. 56); a guide for life for the believer (1966, p. 56); firmly held but not knowledge-apt (1966, p. 57); adhered to because of a life-long “education” (1980, pp. 85–86); and ultimately not based on “exportable” evidence (1980, p. 85). Wittgenstein writes e.g.: “Reasons [for extraordinary beliefs] look entirely different from normal reasons. They are, in a way, quite inconclusive” (1966, p. 56). And: “Life can educate one to a belief in God. ... e.g. sufferings of various sorts. ... Life can force this concept on us” (1980, p. 86).

Moreover Wittgenstein seems willing to consider at least some religious believers his peers of sorts: “In former times people went into monasteries. Were they stupid or in-sensitive people?” (1980, p. 49). And yet, some religious beliefs strike him as “altogether absurd”—as if someone said “2 and 21 is 13” (1966, p. 62). Wittgenstein also regards it as an issue of contingency whether one ends up with a secular or a religious worldview:

Isn’t this altogether like the way one can instruct a child to believe in a God, or that none exists, and it will accordingly be able to produce apparently telling grounds for the one or the other? (1969, §107)

And how can I know what would seem to me to be the only acceptable picture of the world order in case I lived completely differently? I cannot answer this question. (1999, p. 75).

Perhaps one could convince someone that God exists by means of a certain kind of upbringing, by shaping his life in such and such a way (1980, p. 85).

What does all this add up to? In trying to distill a distinctive message from these seemingly illusive aphorisms, it is helpful to draw on Bas van Fraassen’s concept of a “stance” (2002). A stance is a pragmatic whole of beliefs, attitudes, commitments to values, emotions, preferred metaphors, and ways of representing. Examples of stances are empiricism, materialism, scientific secularism, and religious faiths. It is most important for our context that there are limits to rational argument between different stances. This is because some evidence for the beliefs of a stance can be accessed only by those who have already adopted this stance. That is, some evidence is stance-internal.

Now, if we think of B3 as “a culmination” of a specific stance, say, Catholicism, then in order to disagree with B3 the secularist has to reject not just B3 but a large number of further beliefs and other commitments. The disagreement over B3 invariably becomes “systematic”: it concerns many ingredients of the stances; it is spread out in space and time; and it is entrenched (Goldberg 2013). And it involves much more than just beliefs. And yet, it need not end up being “messy” in Elga’s technical sense (2007). That is to say, it need not end up forcing the disagreeing parties to demote each other from the status of peers. Nevertheless, for the secularist to adopt B3 will likely involve a process of “conversion” (van Fraassen 2002, p. 67): there is no neutral line of argument that will rationally force the secularist to adopt B3.

As we saw, Wittgenstein does not think that a difference in stances (secular versus religious) forces us to demote the other. Nor does he think that, in the case of religious disagreements, it is possible to fully disclose one’s evidence. This is one respect in which Wittgenstein differs from Feldman’s (2007) analysis of religious disagreements. But Wittgenstein parts company from Feldman also in rejecting the option of suspending judgment in the face of religious disagreement. As Wittgenstein sees it, in the case of extraordinary beliefs, suspension of belief just is not an option we are able to choose.

It seems inevitable to admit that Wittgenstein’s overall position concerning religious c-disagreements leads again in a relativistic direction. This should not come as a surprise given that van Fraassen’s philosophy of stances is itself naturally classified as relativistic. We might call this position a *relativism of stances*. It differs from *cultural relativism* in being more specific (a stance is less than a whole culture); it differs from *relativism of reasonable belief* in not being restricted to situations in which reasonableness is shared; and it differs from *epistemic relativism* in including a much wider spectrum of elements (than just beliefs and epistemic principles).

In summary, the position comes to this. Sometimes the believer is a ‘stance-dependent peer’—not because her view is as likely to be correct as my own, but because she acts fully rationally (by my standards) in many ways and domains. Sometimes we are able to share much of our evidence, even though some important evidence remains stance-internal and cannot be fully shared. And in some such cases it is then a rationally permissible response to both stick to one’s own view, and to count the believer as rationally entitled to hers. This involves one in recognizing the stance-dependence of one’s own and the other’s views. Note that on this view, the religious person might be an epistemic peer for the non-believer despite the fact that some of her religious pronouncements strike the latter at least initially as absurd. I emphasize that expression “some believer can be peers”: Wittgenstein’s position also leaves room for believers that cannot be made sense of by non-believers or for believers that the non-believers cannot take to be intellectual peers in a relevant sense. In the first case there is no disagreement, in the second there is no peer-disagreement.

## Summary

This paper has tried to explore Wittgensteinian certainties in the context of the peer-disagreement literature. My preliminary and tentative results can be summed up as follows. In some cases, what seems like a peer disagreement over a certainty is merely a disagreement on how certain words are to be used. Once this verbal difference is removed, no real disagreement remains. Wittgenstein briefly alludes to this possibility where he suggests that the difference between us and the Odd Woodsellers may be based on nothing deeper than a different understanding of the words “equal value” or “pile”. But Wittgenstein also considers more substantive c-disagreements. In many of these disagreements, we are forced to demote our interlocutor: agreeing with our certainties is part of the definition of being an intellectual peer. As we saw above, cases in point are the person in our culture who denies that 2 + 2 = 4; someone denying that I have two hands; or the epistemic skeptic. And yet there are also scenarios where Wittgenstein thinks demoting is not obligatory or permissible. Interestingly enough, in some such scenarios, Wittgenstein—or at least my interpretation of *On Certainty*—also makes room for different kinds of relativistic responses. Different forms of relativism surface concerning the question which social institution to adopt, regarding scientific cornerstones, and with respect to religious stances. It is thus a central result of this paper that to make sense of different forms of disagreements over certainties we need different conceptions of peerhood (ordinary, distant, scientific, stance-dependent) and different versions of relativism (cultural, of reasonableness, epistemic and stance-dependent).

It remains for me to relate my results to the study that initially inspired it: Fogelin’s “The Logic of Deep Disagreement” (1985). Fogelin speaks of a “deep disagreement” when the two or more parties to a dispute clash over the truth of their respective but differing “framework propositions”; when the disagreement persists “even when normal criticisms have been answered”; when the disagreement is “immune to appeals to facts”; or when two or more “forms of life” are in conflict (1985, p. 8). Fogelin was obviously inspired by Wittgenstein, and he quotes extensively from *On Certainty*.

I have tried to take Fogelin’s ideas forward by using more refined and detailed conceptualizations for the phenomena Fogelin was connecting: disagreements and certainties. Whereas Fogelin worked with a simple dichotomy of deep and shallow disagreements, I have relied on the distinctions made prominent in the epistemology of peer disagreement. And whereas Fogelin treated all certainties as falling in one and the same category of “framework propositions”, I have used a taxonomy of certainties that distinguishes between five broad epistemic categories. The payoff of my refinements is—or may eventually be when all this is developed in much greater detail—a more fine-grained understanding of when argumentation, in the sense of rational persuasion, is possible, and when it is bound to fail. Such work will also give us a better grasp of what kinds of disagreement lend themselves to motivating forms of relativism.

## Footnotes

1 The most recent contribution is Pritchard (2018). And there are many others in between that raise related issues.

2 See Kusch (2011a, b, c; 2012; 2013; 2014a, b; 2015a, b; 2016a, b; 2017a, b, c; 2018).

3 There are of course many alternatives to the view set out in the above. See especially Brice (2014), Coliva (2010), McGinn (1989), Moyal-Sharrock (2007), Pleasants (2008), Pritchard (2016, 2018), Schönbaumsfeld (2016), and Stroll (1994). It seems to me that my detailed criticism of McGinn and Moyal-Sharrock in Kusch (2016b), and Pritchard in Kusch (2018), also undermines the readings offered by other authors.—My own rendering is close to Williams (2005) and Wright (2004).

4 One of my referees suggested that I consider the Amazonian tribe of the Piraha in this context. The Piraha do not have numerals at all. Everett (2012) includes a fascinating account of this tribe’s language and culture. Everett’s general approach seems to me to cohere with Wittgenstein’s discussion of the Odd Woodsellers. Like Wittgenstein, Everett makes every effort to explain why the lack of numerals makes perfect rational sense given the Pirahas’ culture and social structure. Everett seeks to show that the Pirahas act *as we would act* if we were in their circumstances. In my words, he treats the Pirahas as “distant peers”.
